# Meta-Analysis of Clinical Trials Comparing Cefazolin to Cefuroxime, Ceftriaxone, and Cefamandole for Surgical Site Infection Prevention

**DOI:** 10.3390/antibiotics11111543

**Published:** 2022-11-03

**Authors:** Nehad J. Ahmed, Abdul Haseeb, Ahmad Alamer, Ziyad S. Almalki, Abdullah K. Alahmari, Amer H. Khan

**Affiliations:** 1 Department of Clinical Pharmacy, College of Pharmacy, Prince Sattam Bin Abdulaziz University, Alkharj 11942, Saudi Arabia; 2 Discipline of Clinical Pharmacy, School of Pharmaceutical Sciences, Universiti Sains Malaysia, Pulau Pinang 11800, Malaysia; 3 Clinical Pharmacy Department, College of Pharmacy, Umm Al-Qura University, Mekkah 13174, Saudi Arabia

**Keywords:** cefamandole, cefazolin, ceftriaxone, cefuroxime, clinical trials, prophylaxis, surgical site infections

## Abstract

Surgical site infections are among the most prevalent and costly healthcare-associated infections, resulting in poor patient outcomes and even death. Cefazolin is a first-generation cephalosporin antibiotic that is widely used for surgical prophylaxis in a variety of surgical disciplines. Although previous studies showed that cefazolin is effective in preventing surgical site infections, other agents, such as cefuroxime and ceftriaxone, were used excessively for surgical patients. The present analysis included only clinical trials comparing the efficacy of cefazolin to cefuroxime, ceftriaxone, and cefamandole in lowering SSIs using PubMed, Google Scholar, and ClinicalTrials.gov. Review Manager software (RevMan version 5.4) was used to conduct the meta-analyses. A total of 12,446 patients were included in the study. Among these patients, 6327 patients received cefazolin and 6119 patients received cefamandole, cefuroxime, or ceftriaxone. Our analysis showed that cefazolin is as effective as cefuroxime, cefamandole, and ceftriaxone in preventing surgical site infections. Hence, our findings have provided evidence for the use of cefazolin before surgeries because of its efficacy, as previous studies showed that it is inexpensive and safer than other agents.

## 1. Introduction

The surgical site infection (SSI) appears in a wound created by a surgical or post-operative procedure of any cavity, joint, bone, tissue, or prosthesis involved [1]. SSI is considered if it occurs within 30 days of the operation or within 90 days if prosthesis implantation is involved, and is classified according to the tissues involved into superficial incisional, which involves only skin or subcutaneous tissue at the site of incision; deep incisional, which covers deep soft tissues (fasciae and muscles); and infections in organs and spaces, which involves any part of the anatomy other than the incision that was opened or manipulated during the operation [2,3]. These infections are among the most prevalent and costly healthcare-associated infections, resulting in poor patient outcomes and even death [4].

It is generally acknowledged that antibiotic prophylaxis is necessary for clean-contaminated, contaminated, and unclean wounds [5]. One antibiotic dose is typically enough if the procedure lasts four hours or less; however, further antibiotic doses may be needed to maintain the concentration, especially if the antibiotic has a short half-life [6]. Repeat intraoperative antibiotic administration is necessary when the surgery lasts more than four hours or there is an expected blood loss of more than 1500 mL. Unless there is a known infection, prophylactic antibiotics should be stopped within 24 h [7].

Cephalosporins are beta-lactam antimicrobials used to manage a wide range of infections from Gram-positive and Gram-negative bacteria. First-generation cephalosporins, such as cefazolin, cefadroxil, cephalothin, cephradine, cephapirin, and cephalexin, have active coverage against most Gram-positive cocci, such as *streptococci* spp. and *staphylococci* spp., while having minimal coverage against Gram-negative bacteria [8]. Second-generation cephalosporins include cefuroxime, cefmetazole, cefprozil, cefoxitin, and cefotetan. Second-generation cephalosporins have less activity against Gram-positive cocci than first-generation cephalosporins; however, they have increased activity against Gram-negative bacilli. Third-generation cephalosporins include cefotaxime, cefdinir, ceftazidime, ceftriaxone, cefixime, and cefpodoxime. Third-generation cephalosporins have less coverage against most Gram-positive organisms; however, they have increased coverage against *Enterobacteriaceae*, *Neisseria* spp., and *Haemophilus influenzae* [8].

Cefazolin is a first-generation cephalosporin antibiotic that is widely used for surgical prophylaxis in a variety of surgical disciplines [9]. Because of its superior safety profile, low cost, and targeted activity against germs typically encountered during surgical operations, it remains the medication of choice for surgical prophylaxis in many procedures [10]. According to Geroulanos et al., cefazolin has been frequently recommended with success in surgical prophylaxis, although broad-spectrum cephalosporins such as ceftriaxone are generally not recommended [11].

Maki et al. stated that studies and findings from the 1990s show that the first-generation cephalosporin cefazolin is just as effective as second-generation cephalosporins, such as cefamandole or cefuroxime, in preventing surgical site infections [12]. Surat et al. noted that a new local perioperative antibiotic prophylaxis guideline set first-generation cefazolin as the new standard prophylactic antibiotic rather than second-generation cefuroxime [13]. As a result, we decided to identify clinical trials, pool the data, and conduct a meta-analysis to compare the efficacy of cefazolin in reducing SSIs to cefuroxime, ceftriaxone, and cefamandole, which were routinely used to prevent postoperative site infections.

## 2. Materials and Methods

The analysis included the clinical trials that compare the efficacy of cefazolin to cefuroxime, ceftriaxone, and cefamandole in lowering SSIs that were identified using PubMed, Google Scholar, and ClinicalTrials.gov. The keywords “surgical site infections” AND “cefazolin” AND “ceftriaxone” OR “cefuroxime” OR “cefamandole” were used in the search.

The analysis comprised only published human clinical studies and included all of the trials that were published from 1976 to 2022. The study excludes other sorts of studies. The data obtained comprised the overall number of surgical patients who had cefazolin as well as the number of surgical site infections in these patients. In addition to that, the study included the total number of surgical patients who had cefuroxime, ceftriaxone, or cefamandole, and the number of surgical site infections that occurred in these patients.

The occurrence of surgical site infections in the cefazolin and other cephalosporin groups was the endpoint of our study. The occurrence of surgical site infections was compared between cefazolin and cefuroxime; between cefazolin and ceftriaxone; and between cefazolin and cefamandole. Following that, we divided the patients into two groups: one with cefazolin; and one with cefuroxime, ceftriaxone, and cefamandole to assess the rate of surgical site infection between cefazolin and the other agents in general.

An odds ratio and a random effect model with 95% confidence intervals were used to compare the groups. A funnel plot was used to assess publication bias. The data were shown by creating a forest plot with OR. The I^2^ statistic was used to examine the studies’ heterogeneity. An I^2^ score of 50% or higher indicated significant trial heterogeneity. A statistically significant p-value of 0.05 was used. Review Manager software (RevMan version 5.4) was used to conduct the meta-analyses.

## 3. Results

**Figure 1:**
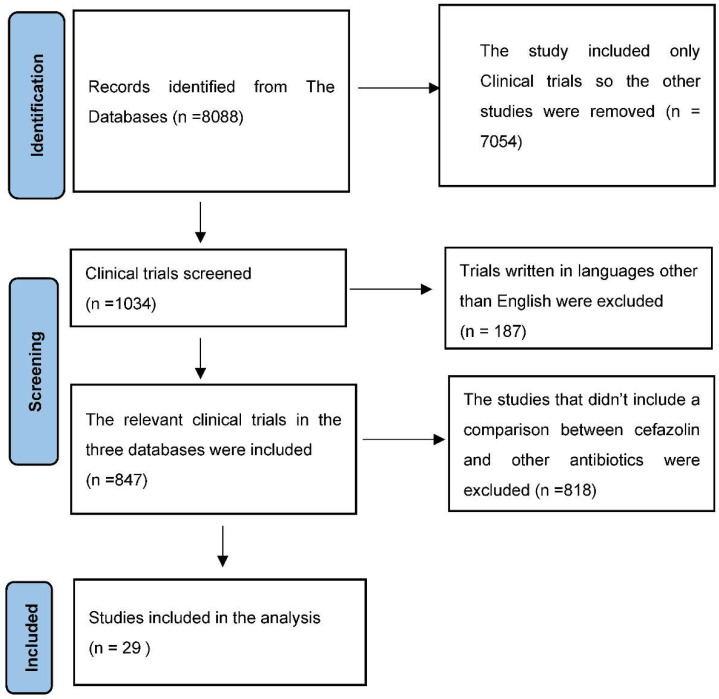
Study Flow Chart.

The present analysis included twenty-nine studies. All of the included trials were clinical trials. Four studies compare cefazolin with cefamandole; eleven studies compare cefazolin and ceftriaxone; eleven studies compare cefazolin with cefuroxime; and three compare cefazolin with both cefuroxime and cefamandole (Table 1). A total of 12,446 patients were included in the study. Among these patients, 6327 patients received cefazolin and 6119 patients received cefamandole, cefuroxime, or ceftriaxone (Figure 1).

**Figure 2:**
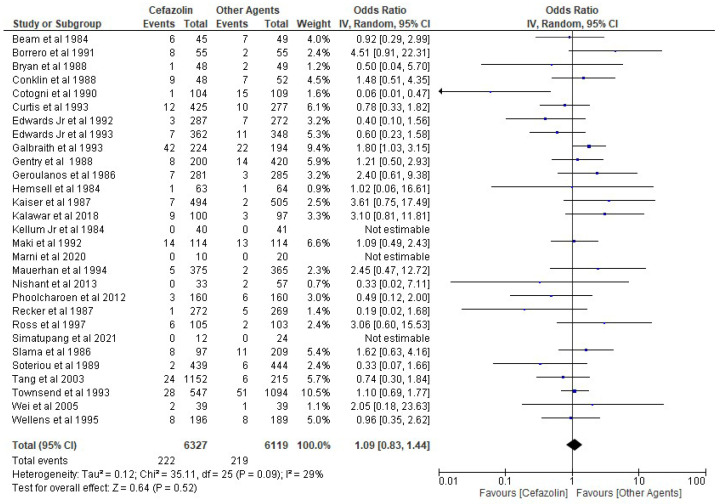
The rate of SSIs in the cefazolin group vs. other cephalosporins. The included studies were Beam et al. 1984 [39], Borrero et al. 1991 [29], Bryan et al. 1988 [32], Conklin et al. 1988 [33], Cotogni et al. 1990 [30], Curtis et al. 1993 [24], Edwards Jr et al. 1992 [28], Edwards Jr et al. 1993 [25], Galbraith et al. 1993 [26], Gentry et al. 1988 [34], Geroulanos et al. 1986 [37], Hemsell et al. 1984 [40], Kaiser et al. 1987 [35], Kalawar et al. 2018 [16], Kellum Jr et al. 1984 [41], Maki et al. 1992 [12], Marni et al. 2020 [15], Mauerhan et al. 1994 [23], Nishant et al. 2013 [17], Phoolcharoen et al. 2012 [18], Recker et al. 1987 [36], Ross et al. 1997 [21], Simatupang et al. 2021 [14], Slama et al. 1986 [38], Soteriou et al. 1989 [31], Tang et al. 2003 [20], Townsend et al. 1993 [27], Wei et al. 2005 [19], Wellens et al. 1995 [22].

The results of the present analysis showed that the rate of SSIs in the cefazolin group was 3.51% and the total rate of SSIs in the other cephalosporins group was 3.58%. The present meta-analysis showed these differences to be statistically not significant using the random-effect model (OR 1.09, 95% CI 0.83–1.44, *p*-value more than 0.05) (Figure 2).

**Figure 3:**
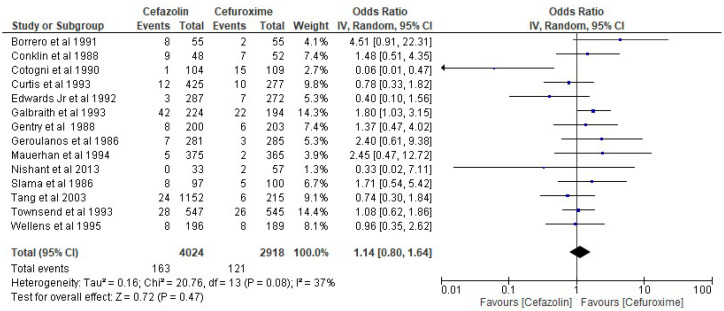
The rate of SSIs in the cefazolin group vs. cefuroxime group. The included studies were Borrero et al. 1991 [29], Conklin et al. 1988 [33], Cotogni et al. 1990 [30], Curtis et al. 1993 [24], Edwards Jr et al. 1992 [28], Galbraith et al. 1993 [26], Gentry et al. 1988 [34], Geroulanos et al. 1986 [37], Mauerhan et al. 1994 [23], Nishant et al. 2013 [17], Slama et al. 1986 [38], Tang et al. 2003 [20], Townsend et al. 1993 [27], Wellens et al. 1995 [22].

The results of the present analysis showed that the rate of SSIs in the cefazolin group was 4.05% and the total rate of SSIs in the cefuroxime group was 4.15%. The present meta-analysis showed these differences to be statistically not significant using the random-effect model (OR 1.14, 95% CI 0.80–1.64, *p*-value more than 0.05) (Figure 3).

**Figure 4:**
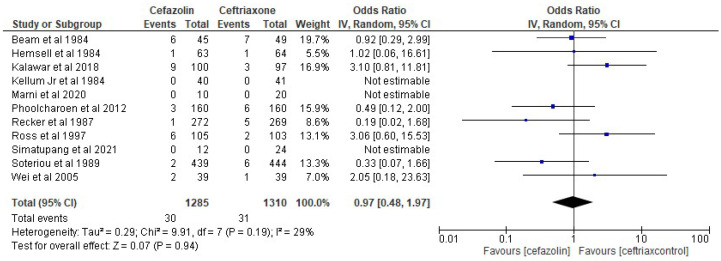
The rate of SSIs in the cefazolin group vs. ceftriaxone group. The included studies were Beam et al. 1984 [39], Hemsell et al. 1984 [40], Kalawar et al. 2018 [16], Kellum et al. 1984 [41], Marni et al. 2020 [15], Phooicharoen et al. 2012 [18], Recker et al. 1987 [36], Ross et al. 1997 [21], Simatupang et al. 2021 [14], Soteriou et al. 1989 [31], Wei et al. 2005 [19].

The results of the present analysis showed that the rate of SSIs in the cefazolin group was 2.33% and the total rate of SSIs in the ceftriaxone group was 2.37%. The present meta-analysis showed these differences to be statistically not significant using the random-effect model (OR 0.97, 95% CI 0.48–1.97, *p*-value more than 0.05) (Figure 4).

**Figure 5:**
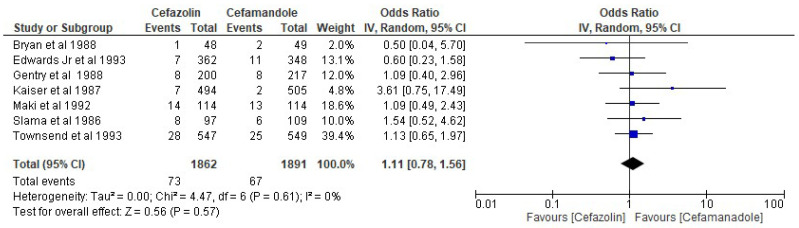
The rate of SSIs in the cefazolin group vs. cefamandole group. The included studies were Bryan et al. 1988 [32], Edwards Jr et al. 1993 [25], Gentry et al. 1988 [34], Kaisar et al. 1987 [35], Maki et al. 1992 [12], Slama et al. 1986 [38], Townsend et al. 1993 [27].

The results of the present analysis showed that the rate of SSIs in the cefazolin group was 3.92% and the total rate of SSIs in the cefamandole group was 3.54%. The present meta-analysis showed these differences to be statistically not significant using the random-effect model (OR 1.11, 95% CI 0.78–1.56, *p*-value more than 0.05) (Figure 5).

**Figure 6:**
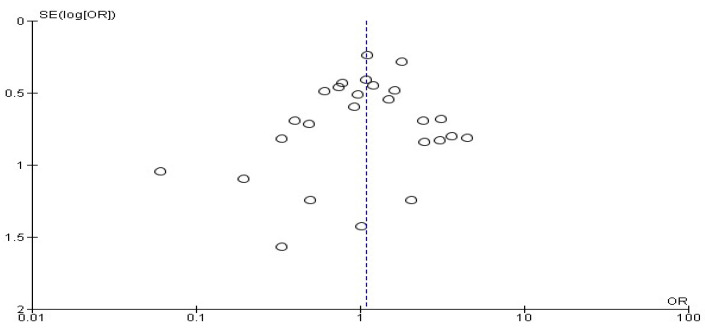
Funnel plot (surgical site infections rate).

The heterogeneity test *p*-value and the *p*-value of the overall effect test showed non-significant results in the present study. Moreover, our funnel plot shows the symmetrical distribution of the study, which suggests a low level of publication bias (Figure 6). So, the findings of the present study findings have good validity.

## 4. Discussion

Postoperative healthcare-related infections, particularly surgical site infections, are associated with worsening general health status, and a greater social and economic cost [42,43]. An evidence-based approach is thought to prevent more than half of HAIs and perioperative antibiotic treatment may play a key role in infection prevention. Cefazolin is most commonly used for surgical prophylaxis in patients who do not have a history of beta-lactam allergy or methicillin-resistant Staphylococcus aureus infection [44,45,46].

The results of our meta-analysis show that cefazolin is as effective as cefuroxime and cefamandole in preventing surgical site infections. Edwards Jr reported that cefazolin is still the best cost-effective antibiotic for prophylaxis in clean vascular surgical procedures [25]. According to Townsend et al., the locations of infection and level of tissue involvement were not substantially different among the cefamandole, cefazolin, and cefuroxime groups. Because no differences in effectiveness in avoiding postoperative site infections were proven in a properly planned experiment, the drug costs, including the costs of preparation and delivery, may be the only criteria used to choose between these three antibiotic prophylaxis regimens [27]. Furthermore, Edwards Jr 1992 stated that the trend in infection rates implies that cefazolin is more effective than cefuroxime for perioperative prophylaxis, despite the fact that the difference was not statistically significant [28]. Wellens et al. reported that short-term treatment of 3 g cefazolin or cefuroxime failed to establish a therapeutic advantage of one antibiotic over the other in terms of clinical outcome, incidence or site of infection, or organisms detected [22]. When compared to the first-generation cephalosporin, cefazolin, Curtis et al. found that the second-generation cephalosporin, cefuroxime, did not reduce the incidence of wound infection. Because institutional antibiotic purchase and administration costs vary, careful consideration of these aspects will allow the best cost-effective infection prophylaxis strategy in cardiac surgery to be determined [24]. According to Nishant et al., there was no difference in the occurrence of surgical site infection between cefazolin and cefuroxime [17]. Furthermore, Gentry et al. stated that there are no differences in the efficacy of cefuroxime, cefamandole, and cefazolin in avoiding postoperative infections in patients undergoing open-heart surgery [35].

The results of our meta-analysis show that cefazolin is as effective as ceftriaxone in preventing surgical site infections. Phoolcharoen et al. found no difference in reducing infectious morbidity between single-dose preoperative ceftriaxone and cefazolin in patients having a hysterectomy [18]. According to Kalawar et al., there is no difference in the efficiency of cefazolin and ceftriaxone in the prevention of surgical site infection [16]. Simatupang et al. demonstrated that cefazolin and ceftriaxone have the same effectiveness in avoiding germ growth in surgical wounds [14]. Furthermore, Ross et al. stated that ceftriaxone is therapeutically equivalent to cefazolin in preventing postoperative wound infections in patients who had peripheral vascular surgery [21]. Marni et al. found no difference in the prevention of postoperative infections between ceftriaxone and cefazolin [15]. According to Wei et al., there is no statistically significant difference in the incidence of peritonitis and wound infection between the ceftriaxone and cefazolin groups [19].

Current studies reported that cefazolin is the recommended antibiotic for most surgical procedures. Jocum et al. reported that cefazolin is utilized for prophylaxis in most surgical operations. It has been carefully researched and has proved efficacy [47]. According to Ahmed et al., cefazolin is the antibiotic of choice for prophylaxis in the majority of surgeries because it has been widely investigated and has established efficacy [48]. Wolfhagen et al. stated that cefazolin is the most commonly indicated drug for surgical antibiotic prophylaxis [49]. Furthermore, according to Isserman et al., cefazolin, a first-generation cephalosporin, is the most often recommended antibiotic for perioperative prophylaxis to decrease surgical site infections [50]. Cefazolin has been used in clinical practice for about 40 years, according to Kusaba, and a considerable body of research has been collected; in addition, its efficacy and safety are well-established when compared to other antibiotics. As a result, cefazolin has been selected as a first-line anti-microbial for prophylaxis following several surgical operations, such as cardiovascular surgery, hysterectomy, and arthroplasty [51]. Bratzler et al. stated that because of its favorable safety profile, low cost, and focused activity against germs typically encountered during surgical operations, cefazolin remains the medication of choice for surgical prophylaxis in numerous procedures [10]. Furthermore, Alemkere stated that the American Society of Health-System Pharmacists recommends ceftriaxone only for high-risk biliary tract procedures and colorectal surgery. Because ceftriaxone is a broad-spectrum medicine, it is more likely to produce an emergency of resistance than cefazolin and other commonly used surgical preventive agents [52].

Marano et al. reported that using antimicrobials such as cefazolin and cefuroxime during esophagogastric surgery to reduce infections within the intraoperative period should not exceed two antimicrobial agents, at the lowest efficacious and safe doses to avoid the emergence of multi-drug resistance [53]. They reported that further studies are needed to investigate the optimal antimicrobial regimen in esophageal surgery [53]. Ruol et al. stated that a single-dose prophylactic regimen provides adequate prophylaxis and significant cost-savings in comparison with multiple-dose prophylactic regimens in patients undergoing major surgery for esophageal cancer [54]. Mohri et al. stated that cefazolin is a first-generation cephalosporin that has been suggested for antimicrobial prophylaxis in gastric surgery and that a single dose of cefazolin or ampicillin–sulbactam is just as effective as numerous doses in preventing surgical-site infections after gastric cancer surgery [55]. According to the Antibiotic Expert Group, cefazolin is the most commonly recommended antimicrobial for surgical prophylaxis due to a variety of factors, such as its narrow Gram-positive and Gram-negative spectrum coverage for common pathogens, efficacy, safety profile, and low cost [56]. So, cefazolin is recommended as a single agent for most surgeries; however, for some surgeries such as colon surgeries, it is combined with another agent that is active against anaerobic pathogens. Ierano et al. stated that metronidazole is prescribed for some surgeries in addition to cefazolin to give a longer coverage of anaerobic microorganisms [57].

The first limitation of the study was that the timing and route of antibiotic administration were not standardized for all the trials included in the meta-analysis. The second limitation was that the study included old trials due to the small number of recent surgical prophylactic clinical trials. The third limitation was that in some studies, the total number of cases in the intervention and control groups is much different. This would affect the odd ratio comparisons.

## 5. Conclusions

Our analysis showed that cefazolin is as effective as cefuroxime, cefamandole, and ceftriaxone in preventing surgical site infections. Our findings provide evidence for the use of cefazolin before surgeries because it has a similar efficacy to cefuroxime, cefamandole, and ceftriaxone. So, it is recommended to add the first-generation cephalosporin cefazolin instead of ceftriaxone and cefuroxime in the current effective clinical practice guidelines for antimicrobial prophylaxis.

## Figures and Tables

**Table 1:** The studies that were included in the analysis.

Study	Year	Antibiotics
Simatupang et al. [14]	2021	Cefazolin vs. Ceftriaxone
Marni et al. [15]	2020	Cefazolin vs. Ceftriaxone
Kalawar et al. [16]	2018	Cefazolin vs. Ceftriaxone
Nishant et al. [17]	2013	Cefazolin vs. Cefuroxime
Phoolcharoen et al. [18]	2012	Cefazolin vs. Ceftriaxone
Wei et al. [19]	2005	Cefazolin vs. Ceftriaxone
Tang et al. [20]	2003	Cefazolin vs. Cefuroxime
Ross et al. [21]	1997	Cefazolin vs. Ceftriaxone
Wellens et al. [22]	1995	Cefazolin vs. Cefuroxime
Mauerhan et al. [23]	1994	Cefazolin vs. Cefuroxime
Curtis et al. [24]	1993	Cefazolin vs. Cefuroxime
Edwards Jr et al. [25]	1993	Cefazolin vs. Cefamandole
Galbraith et al. [26]	1993	Cefazolin vs. Cefuroxime
Townsend et al. [27]	1993	Cefazolin vs. Cefuroxime vs. Cefamandole
Edwards Jr et al. [28]	1992	Cefazolin vs. Cefuroxime
Maki et al. [12]	1992	Cefazolin vs. Cefamandole
Borrero et al. [29]	1991	Cefazolin vs. Cefuroxime
Cotogni et al. [30]	1990	Cefazolin vs. Cefuroxime
Soteriou et al. [31]	1989	Cefazolin vs. Ceftriaxone
Bryan et al. [32]	1988	Cefazolin vs. Cefamandole
Conklin et al. [33]	1988	Cefazolin vs. Cefuroxime
Gentry et al. [34]	1988	Cefazolin vs. Cefuroxime vs. Cefamandole
Kaiser et al. [35]	1987	Cefazolin vs. Cefamandole
Recker et al. [36]	1987	Cefazolin vs. Ceftriaxone
Geroulanos et al. [37]	1986	Cefazolin vs. Cefuroxime
Slama et al. [38]	1986	Cefazolin vs. Cefuroxime vs. Cefamandole
Beam et al. [39]	1984	Cefazolin vs. Ceftriaxone
Hemsell et al. [40]	1984	Cefazolin vs. Ceftriaxone
Kellum Jr et al. [41]	1984	Cefazolin vs. Ceftriaxone

## Data Availability

Not applicable.
